# Supplementary material for: p53 induces ARTS to promote mitochondrial apoptosis
Source: Cell Death Dis. 2021 Feb 24;12(2):204. doi: 10.1038/s41419-021-03463-8 (PMC7904775; doi:10.1038/s41419-021-03463-8)
Supplement: Supplementary file 1 — Supplementary Information [file 41419_2021_3463_MOESM1_ESM.pdf]

p53 induces ARTS to promote mitochondrial apoptosis

Qian Hao[1,2,#], Jiaxiang Chen[3,†,#], Junming Liao[3], Yingdan Huang[1,2], Yu Gan[4], Sarit Larisch[5], Shelya X Zeng[3], Hua Lu[3,*], and Xiang Zhou[1,2,6,7,*]

[1] Fudan University Shanghai Cancer Center and Institutes of Biomedical Sciences, Fudan University, Shanghai 200032, China

[2] Department of Oncology, Shanghai Medical College, Fudan University, Shanghai 200032, China

[3] Department of Biochemistry & Molecular Biology and Tulane Cancer Center, Tulane University School of Medicine, New Orleans, LA 70112, USA

[4] Department of Physiology, Medical College of Nanchang University, Nanchang 330006, China

[5] Cell Death and Cancer Research Laboratory, Department of Biology, University of Haifa, Haifa 31905, Israel

[6] Key Laboratory of Breast Cancer in Shanghai, Fudan University Shanghai Cancer Center, Fudan University, Shanghai 200032, China

[7] Shanghai Key Laboratory of Medical Epigenetics, International Co-laboratory of Medical Epigenetics and Metabolism, Ministry of Science and Technology, Institutes of Biomedical Sciences, Fudan University, Shanghai 200032, China

Running Title: p53 and ARTS trigger apoptosis in cooperation

[#] Equal contribution

[†] Present address: Department of Physiology, Medical College of Nanchang University, Nanchang 330006, China

[*] Corresponding Authors:

Hua Lu, Department of Biochemistry & Molecular Biology and Tulane Cancer Center, Tulane University School of Medicine, New Orleans, LA 70112, USA. Email: hlu2@tulane.edu

Xiang Zhou, Fudan University Shanghai Cancer Center and Institutes of Biomedical Sciences, Fudan University, Shanghai 200032, P. R. China. Email: xiangzhou@fudan.edu.cn

Supplementary Figure Legends

Figure S1. ARTS interacts with p53 in the mitochondria. The mitochondrial fractions of HCT116 $^{p53+/+}$ cells stably expressing Flag-ARTS treated with or without Etoposide were prepared for the co-IP assay using antibodies as indicated.

Figure S2. (A, B) Overexpression of ARTS augments Cisplatin- or Nutlin-3-induced apoptosis. H460 cells transfected with the control vector or ARTS plasmid were treated with 5 μM Cisplatin or 40 μM Nutlin-3 for 48 h. The apoptosis of the cells were then analyzed by flow cytometry for apoptosis. (C, D) Ablation of ARTS diminishes Cisplatin- or Nutlin-3-induced apoptosis. HCT116 $^{p53+/+}$ cells stably expressing control or ARTS shRNA were treated with 20 μM Cisplatin or 20 μM Nutlin-3 for 48 h and subjected to flow cytometry analysis for apoptosis.

Figure S3. (A) Knockdown of ARTS reduces mitochondrial accumulation of p53 in response to Cisplatin or Nutlin-3 treatment. HCT116 $^{p53+/+}$ cells expressing the control or ARTS shRNA were treated with Cisplatin or Nutlin-3 and subjected to cellular component fractionation. IB assays were performed to assess the expression of p53 and Bcl-XL in the nucleus, cytoplasm and mitochondria, respectively. The expression of COX IV indicates the mitochondrial fraction. (B) Knockout of Bcl-XL via the CRISPR-Cas9 method induces the level of cleaved-PARP in HCT116 $^{p53+/+}$ cells.

Figure S1

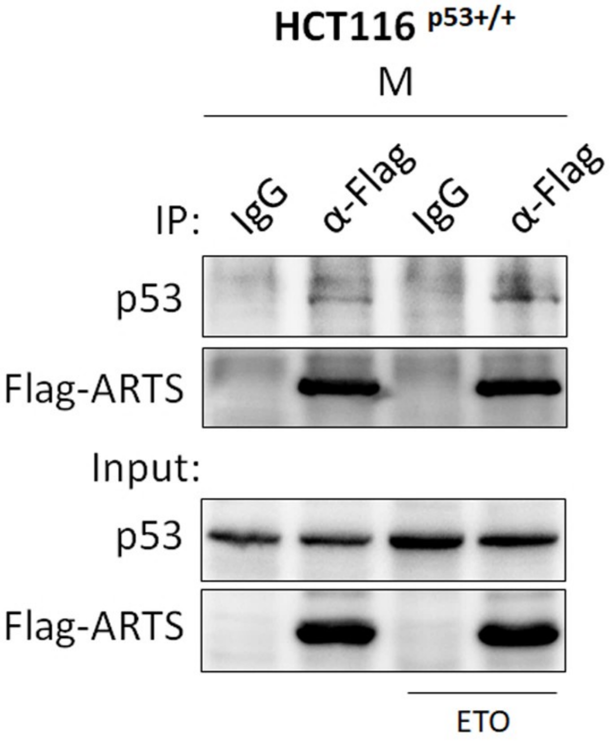
HCT116 p53+/+
M
IP:
IgG
α-Flag
IgG
α-Flag
p53
Flag-ARTS
Input:
p53
Flag-ARTS
ETO

Figure S2

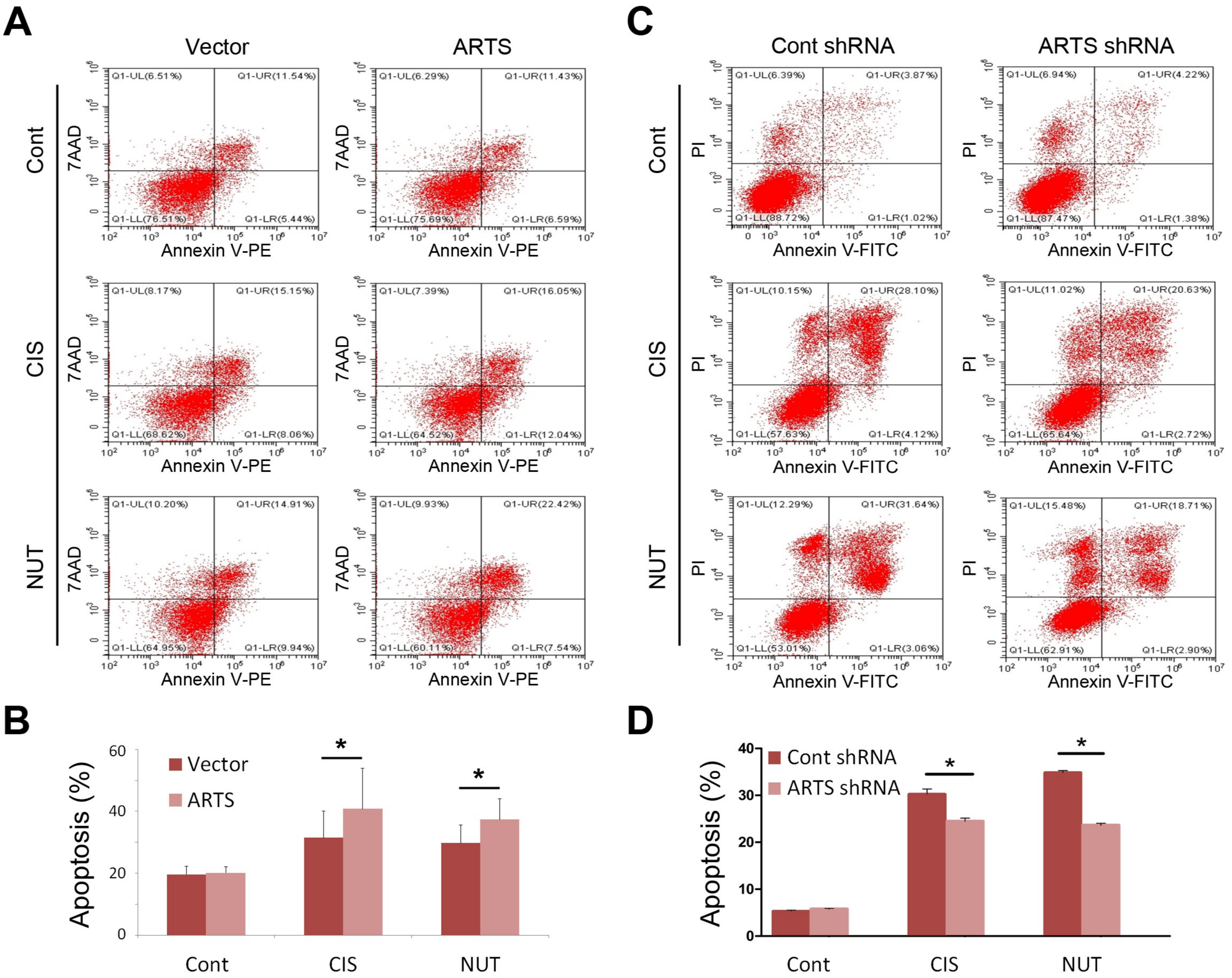
A
Vector
ARTS
Cont
CIS
NUT
7AAD
Annexin V-PE
Q1-UL(6.51%)
Q1-UR(11.54%)
Q1-LL(76.51%)
Q1-LR(5.44%)
Q1-UL(6.29%)
Q1-UR(11.43%)
Q1-LL(75.69%)
Q1-LR(6.59%)
Q1-UL(8.17%)
Q1-UR(15.15%)
Q1-LL(68.62%)
Q1-LR(8.06%)
Q1-UL(7.39%)
Q1-UR(16.05%)
Q1-LL(64.52%)
Q1-LR(12.04%)
Q1-UL(10.20%)
Q1-UR(14.91%)
Q1-LL(64.95%)
Q1-LR(9.94%)
Q1-UL(9.93%)
Q1-UR(22.42%)
Q1-LL(60.11%)
Q1-LR(7.54%)
C
Cont shRNA
ARTS shRNA
PI
Annexin V-FITC
Q1-UL(6.39%)
Q1-UR(3.87%)
Q1-LL(88.72%)
Q1-LR(1.02%)
Q1-UL(6.94%)
Q1-UR(4.22%)
Q1-LL(87.47%)
Q1-LR(1.38%)
Q1-UL(10.15%)
Q1-UR(28.10%)
Q1-LL(57.63%)
Q1-LR(4.12%)
Q1-UL(11.02%)
Q1-UR(20.63%)
Q1-LL(65.64%)
Q1-LR(2.72%)
Q1-UL(12.29%)
Q1-UR(31.64%)
Q1-LL(53.01%)
Q1-LR(3.06%)
Q1-UL(15.48%)
Q1-UR(18.71%)
Q1-LL(62.91%)
Q1-LR(2.90%)
B
Apoptosis (%)
Vector
ARTS
Cont
CIS
NUT
*
D
Apoptosis (%)
Cont shRNA
ARTS shRNA
Cont
CIS
NUT
*

Figure S3

A

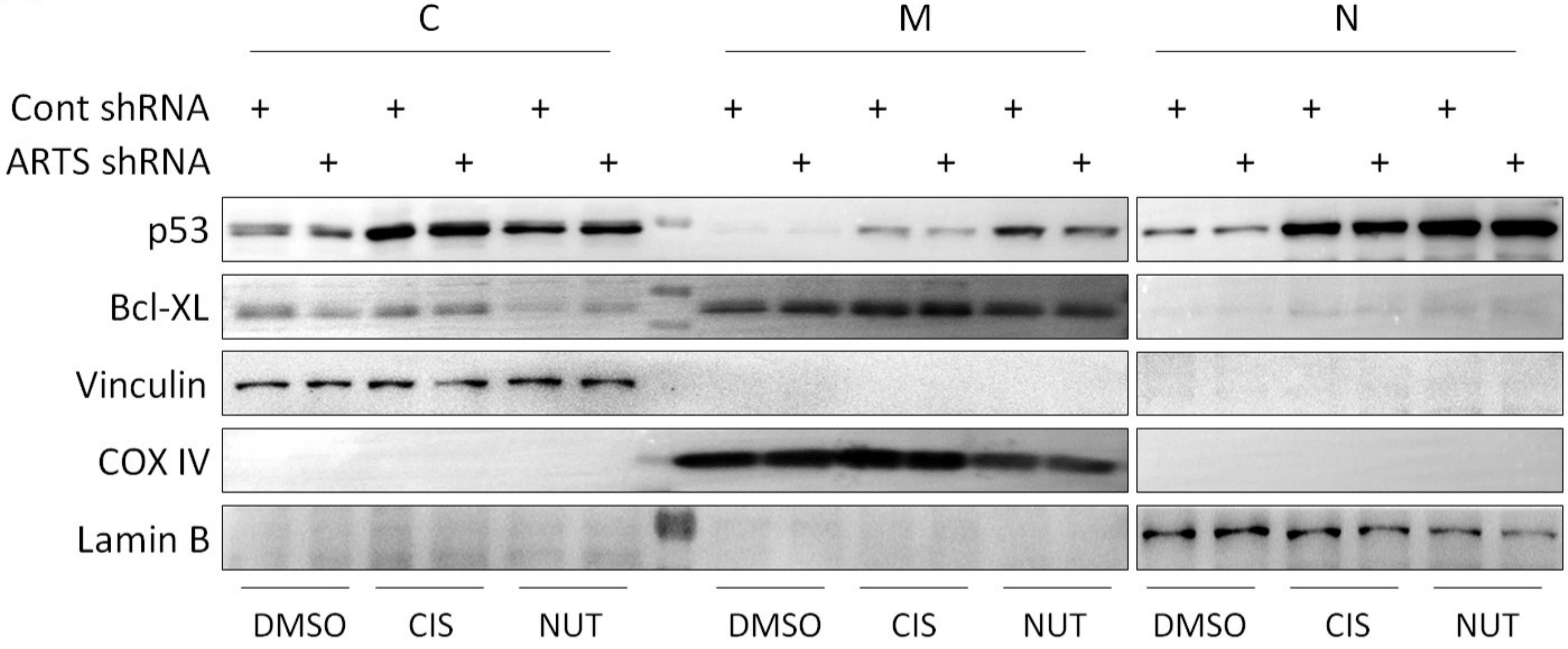
C
M
N
Cont shRNA + + + + + + + + +
ARTS shRNA + + + + + + + + +
p53
Bcl-XL
Vinculin
COX IV
Lamin B
DMSO
CIS
NUT
DMSO
CIS
NUT
DMSO
CIS
NUT

B

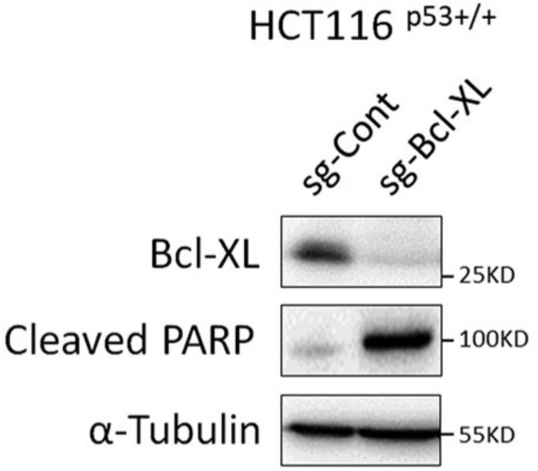
HCT116 p53+/+
sg-Cont
sg-Bcl-XL
Bcl-XL
Cleaved PARP
α-Tubulin
25KD
100KD
55KD
